# Chemotherapy followed by surgery versus surgery alone in patients with resectable oesophageal squamous cell carcinoma: Long-term results of a randomized controlled trial

**DOI:** 10.1186/1471-2407-11-181

**Published:** 2011-05-19

**Authors:** Jurjen J Boonstra, Tjebbe C Kok, Bas PL Wijnhoven, Mark van Heijl, Mark I van Berge Henegouwen, Fiebo JW ten Kate, Peter D Siersema, Winand NM Dinjens, Jan JB van Lanschot, Hugo W Tilanus, Ate van der Gaast

**Affiliations:** 1 Department of Pathology, Josephine Nefkens Institute, Erasmus MC, University Medical Center, Rotterdam, The Netherlands; 2 Department of Surgery, Erasmus MC, University Medical Center, Rotterdam, The Netherlands; 3 Department of Internal Medicine, Maasstad Hospital, Rotterdam, The Netherlands; 4 Department of Surgery, Academic Medical Center, Amsterdam, The Netherlands; 5 Department of Pathology, University Medical Center Utrecht, Utrecht, The Netherlands; 6 Department of Gastroenterology and Hepatology6, University Medical Center Utrecht, Utrecht, The Netherlands; 7 Department of Internal Medicine, Josephine Nefkens Institute, Erasmus MC, University Medical Center, Rotterdam, The Netherlands

## Abstract

**Background:**

This is a randomized, controlled trial of preoperative chemotherapy in patients undergoing surgery for oesophageal squamous cell carcinoma (OSCC). Patients were allocated to chemotherapy, consisting of 2-4 cycles of cisplatin and etoposide, followed by surgery (CS group) or surgery alone (S group). Initial results reported only in abstract form in 1997, demonstrated an advantage for overall survival in the CS group. The results of this trial have been updated and discussed in the timeframe in which this study was performed.

**Methods:**

This trial recruited 169 patients with OSCC, 85 patients assigned to preoperative chemotherapy and 84 patients underwent immediate surgery. The primary study endpoint was overall survival (OS), secondary endpoints were disease free survival (DFS) and pattern of failure. Survival has been determined from Kaplan-Meier curves and treatment comparisons made with the log-rank test.

**Results:**

There were 148 deaths, 71 in the CS and 77 in the S group. Median OS time was 16 months in the CS group compared with 12 months in the S group; 2-year survival rates were 42% and 30%; and 5-year survival rates were 26% and 17%, respectively. Intention to treat analysis showed a significant overall survival benefit for patients in the CS group (*P *= 0.03, by the log-rank test; hazard ratio [HR] 0.71; 95%CI 0.51-0.98). DFS (from landmark time of 6 months after date of randomisation) was also better in the CS-group than in the S group (*P *= 0.02, by the log-rank test; HR 0.72; 95%CI 0.52-1.0). No difference in failure pattern was observed between both treatment arms.

**Conclusions:**

Preoperative chemotherapy with a combination of etoposide and cisplatin significantly improved overall survival in patients with OSCC.

## Background

Oesophageal squamous cell carcinoma (OSCC) accounts for most cases of oesophageal cancer worldwide [1,2]. Even after complete surgical dissection, the prognosis of patients with OSCC is poor, with 5-year survival rates of 20 to 30%. Factors that contribute to this dismal prognosis include presence of locally advanced disease and undetected metastatic cancer at diagnosis. Because of the high rates of locoregional and distant failure, there is much interest in the combination of systemic chemotherapy and local surgical treatment.

The potential benefits of preoperative chemotherapy include increasing the likelihood of curative resection by downstaging the tumour and rapidly improving tumour-related symptoms. It is also been thought that systemic chemotherapy could contribute to the eradication of micro-metastases and circulating tumour cells. More recently, the importance of systemic disease control has been emphasized by new insights in the metastasizing process of cancer [3]. For decades, the dissemination of cancer has been considered the final stage in a deteriorating process. Now, there is accumulating evidence that dissemination already can occur at an early stage of the disease [4]. In theory, the use of preoperative chemotherapy may therefore have a positive impact on survival of patients with oesophageal cancer. Here, we report the design and long-term results of a randomized controlled trial in patients with resectable OSCC, comparing preoperative chemotherapy with cisplatin and etoposide followed by surgery to surgery alone.

## Methods

All eligible patients had histologically confirmed squamous cell carcinoma of the intra-thoracic ooesophagus. Patients were deemed resectable if the disease was clinically limited to the locoregional area (tumour stage 1, 2 or 3; any nodal stage and no metastases). Patients with carcinoma of the distal oesophagus and suspected celiac lymph nodes involvement (M1a) were also considered eligible for surgery. Patients had to be below 80 years of age, in adequate physical condition (Karnofsky score >70) to undergo surgery and had to have adequate hepatic, renal and bone marrow function. Exclusion criteria were synchronous cancer, tumour localization in the cervical ooesophagus (upper border, <18 cm from the incisor teeth), severe cardiovascular or pulmonary disease. Patients with previous malignancies were eligible if more than 5 years had elapsed from diagnosis without evidence of tumour recurrence; exceptions were made for adequately treated basal cell cancer of the skin or carcinoma *in situ *of the cervix. Preoperative work-up included clinical examination, oesophago-gastroscopy with biopsies, chest radiography, external ultrasonography of the cervical and upper abdominal region and computed tomography (CT) of the chest and abdomen. Radionuclide bone scans were performed if indicated. Bronchoscopy was performed when the primary tumour was adjacent to the trachea or main stem bronchus and invasion was suspected.

Central randomisation took place at the Erasmus University Medical Center in Rotterdam (by trial coordinator TCK). Random assignment was stratified by age (<50; 51-60; >60), gender (male; female), weight loss (kg) in the past four months (0-5; 6-10; >10) and length of the tumour (cm) as measured by oesophago-gastroscopy (1-3; 4-6; 7-10; >10). Patients assigned to preoperative chemotherapy were treated with two cycles, followed by a clinical response evaluation. Response evaluation was done three to four weeks after the last cycle of chemotherapy. Clinical response after chemotherapy was evaluated by oesophago-gastroscopy and CT of the chest and abdomen. Tumour responses were assessed according to the World Health Organisation (WHO) criteria [5]. Complete absence of any evidence of malignant disease, including negative biopsies from the former tumour area, was defined as complete response (CR). Partial response (PR) was defined as >50% reduction of tumour bulk, without the appearance of new lesions. Stable disease (SD) was defined as <50% reduction of tumour bulk, without the appearance of new lesions. Progressive disease (PD) was defined as >25% progression of tumour bulk or the appearance of new lesions. Patients with complete or partial responses received two additional courses of chemotherapy, whereas non-responding patients (stable disease or progressive disease) were referred for immediate surgery. Patients with progressive disease (T4 or M1 disease) were treated palliative and observed for survival. Patients, who were randomly assigned to undergo surgery alone, underwent the operation as soon as possible. Patients who received chemotherapy were operated 4 to 6 weeks after the last treatment cycle. The study protocol was approved by the ethics committee of all participating institutions and written informed consent was obtained from all patients.

### Chemotherapy

Cisplatin, at a dose of 80 mg/m^2^, was given intravenously over 4 hours on day one of each cycle preceded and followed by adequate hydration. Etoposide, at a dose of 100 mg/m^2^, was administered intravenously over 2 hours on day 1 (before cisplatin) and day 2, followed by etoposide 200 mg/m^2 ^orally on days 3 and 5. This course was repeated in week 4. In case of clinical response, two subsequent courses of chemotherapy were administered in week 8 and 11. All patients received prophylactic anti-nausea treatment with 5-HT 3 receptor antagonists during chemotherapy. Treatment related toxicities were measured according to the WHO recommendations [5]. Re-treatment with the next cycle was permitted only if the absolute neutrophil count was at least 3,500/mm^3^, and the platelet count was at least 100,000/mm^3^. A delay of treatment of up to 2 weeks was permitted. In patients with severe toxic renal or neurological effects (≥ WHO grade 3) chemotherapy was stopped and patients were referred for surgery.

### Surgery and pathological examination

For carcinomas of the upper half of the intra-thoracic ooesophagus a right-sided thoracotomy was performed. For carcinomas of the lower half of the intra-thoracic ooesophagus a transhiatal oesophagectomy was done. The tumour and its adjacent lymph nodes were dissected *en bloc*. The left gastric artery was transected at its origin, with resection of local lymph nodes. The continuity of the digestive tract was restored by means of gastric tube reconstruction or colonic interposition with a cervical anastomosis. The tumour stage after resection was classified according to the TNM classification of the International Union Against Cancer [6]. Resections were classified as radical when microscopical examination revealed all margins to be free of tumour (R0). Resections were considered not radical, if microscopically examination showed tumour-positive circumferential margin (R1) or presence of macroscopic disease (R2).

### Follow-up

All patients were followed at an interval of three to four months during the first year, every six months for the second year, and annually for up to 5 years post surgery. After 5 years, follow-up data were obtained by telephone from the patient or his/her family practitioner. Recurrence of disease was diagnosed on clinical grounds. However, whenever a relapse was suspected, radiologic, endoscopic, or histologic confirmation was sought for.

Loco-regional disease recurrence was defined as relapse at the primary site including the anastomosis or in regional lymph nodes. Distant disease recurrence was defined as distant lymph node sites or involvement of distant organs including lung, liver, bone, and subcutaneous tissue.

### Statistical analysis

The planned number of patients to be entered in the study was 80 for each treatment arm. With these numbers of patients the statistical power should be sufficient (power = 0.8; significance 0.05) to detect an increase of the median survival from 10 to 18 months.

Overall survival (OS) was calculated from the date of random assignment to date of death from any cause and surviving patients were censored at the date they were last known to be alive. Disease-free survival (DFS) was calculated from a landmark time of 6 months after date of randomisation to allow for the difference in timing of surgery between the two treatment groups [7]. In this analysis, events including macroscopically incomplete resection, local and distant recurrence, and death arising within the first 6 months after random assignment were regarded as events at this landmark time. Survival curves are presented by the Kaplan-Meier method and treatment comparisons are by the log-rank test.

Statistical analyses were performed using the SPSS statistical package (SPSS Inc., Chicago, IL, USA). Hazard ratios (HR) were calculated with the use of a Cox regression model including treatment alone (primary analysis) and after adjustment for baseline stratification factors. Categorical data were compared with the use of chi-square test or Fisher's exact test, with a test for trend over ordered categories. All statistical comparisons were made with two-tailed tests and *P *values < 0.05 were reported as significant.

## Results

**Figure 1:**
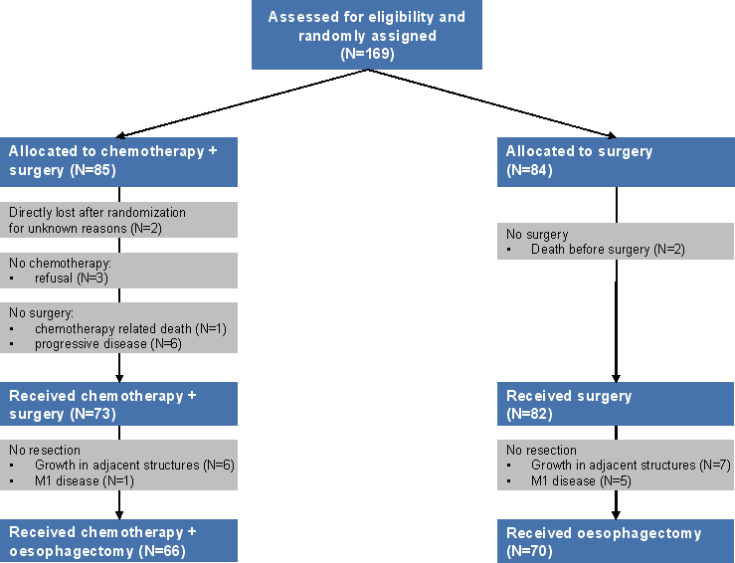
**CONSORT Flow-diagram: random assignment, and compliance to the allocated treatment**. CTX, chemotherapy

Between January, 1989, and January, 1996, 169 patients from six Dutch University Hospitals (Rotterdam, Amsterdam, Utrecht, Groningen, Nijmegen and Maastricht) were randomly assigned to either chemotherapy followed by surgery (CS group, N = 85) or surgery alone (S group, N = 84; Figure 1). An additional number of nine patients were included to adjust for study drop-outs. The majority of patients (N = 122) were included by the Erasmus Medical Center (EMC), Rotterdam. From all participating centers, the EMC is the only hospital that collected outcome data (prospectively) for all patients with oesophageal cancer referred in time this study was performed. Between January, 1989, and January, 1996, 257 patients with OSCC were referred to the EMC. Of these, 183 patients were deemed eligible for surgical resection, of which 122 (67%) were included in this trial. The reasons why 61 (33%) patients were not randomized for this trial are not well documented.

Table 1 shows that the two groups were similar in terms of age, sex, and performance status. Distribution according to weight loss and size of the tumour was also balanced. One patient, allocated to preoperative chemotherapy, had a tumour located in the cervical part of the oesophagus (the reason why this patient was included and randomized remains unclear, even after retrospective analysis of the patient's record). Preoperative staging by CT of the chest and the upper abdomen was performed in 149 patients (88%); two patients (1%) died before the planned CT scan; six patients (4%) were staged by endoscopic ultrasound, external ultrasonography of the cervical and upper abdominal region and chest radiography. From twelve patients (7%) no additional information on preoperative staging was available.

**Table 1:** Patient's characteristics

	Total		CS group		S group		
Characteristics	(N = 169)		(N = 85 )		(N = 84)		*P*-value*
**Age, years**							0,73
<50	31	(18%)	17	(20%)	14	(17%)	
51-60	54	(32%)	25	(29%)	29	(34%)	
>60	84	(50%)	43	(51%)	41	(49%)	
Median	60		60		60		
Range	35 - 79		35 - 76		37 - 79		

**Sex**							0,9
Male	126	(75%)	63	(74%)	63	(75%)	
Female	43	(25%)	22	(26%)	21	(25%)	

**Weight loss (% of normal weight)**							0,24
<5	56	(33%)	30	(35%)	26	(31%)	
6-10	40	(24%)	16	(19%)	24	(29%)	
>10	51	(30%)	30	(35%)	21	(25%)	
Not recorded	22	(13%)	9	(11%)	13	(15%)	

**Tumor length (cm)**							0,17
<3	27	(16%)	14	(17%)	13	(16%)	
4-6	69	(41%)	36	(42%)	33	(39%)	
7-10	55	(32%)	22	(26%)	33	(39%)	
>10	6	(4%)	5	(6%)	1	(1%)	
Not recorded	12	(7%)	8	(9%)	4	(5%)	

**Location of the tumor**							0,66
Cervical	1	(1%)	1	(1%)	0		
Upper third	7	(4%)	3	(3%)	4	(5%)	
Middle third	76	(45%)	38	(45%)	38	(45%)	
Distal third	71	(42%)	34	(40%)	37	(44%)	
Not recorded	14	(8%)	9	(11%)	5	(6%)	

Karnofsky score*							0,53
70 - 80	125	(74%)	60	(71%)	65	(77%)	
90 - 100	38	(22%)	21	(24%)	17	(20%)	
Not recorded	6	(4%)	4	(5%)	2	(3%)	

Abbreviations: *CS, Chemotherapy followed by surgery; S, Surgery alone*

* Comparisons were made by the chi-square test

### Chemotherapy

Of the 85 patients assigned to preoperative chemotherapy, 80 (94%) received chemotherapy; 75 (88%) patients had two or more cycles and 5 patients (13%) received one cycle. The reasons why no chemotherapy or only one cycle was given were patient's refusal (N = 3), death (N = 1), tumour bleeding (N = 3) and renal toxicity grade III (N = 1). Two patients allocated to preoperative chemotherapy, were directly lost to follow-up after randomization. Tracing back the original patient's files was impossible; therefore, it is not clear if these two patients truly received chemotherapy followed by surgery.

Clinical response evaluation after two cycles of chemotherapy showed 43 patients with stable or progressive disease. Partial response to chemotherapy was observed in 32 patients. Of these, 30 patients received two additional cycles of chemotherapy; one received one additional cycle and one had three additional cycles of chemotherapy. Clinical response evaluation after the additional cycles of chemotherapy showed six patients with complete response; 20 patients had partial response; five showed stable disease and one had progressive disease.

Detailed data on chemotherapy related toxicity is not available. In the prior phase II trial a high rate of grade III and IV nausea (38%) and vomiting (20%) was observed, probably due to the fact that 5-HT3 receptor blockers were rarely given throughout the study period [8]. All patients in the current trial received prophylactic anti-nausea treatment with 5-HT 3 receptor antagonists during chemotherapy. No grade III or IV nausea and vomiting were observed. The major non-hematological toxicity (grade III) was alopecia. Hematological toxicity grade III was observed in 23 patients (one renal, twenty-two hematological). Eight patients had grade IV hematological toxicity.

### Outcome of surgery

Surgery was performed in 76 CS and 82 S patients (Table 2). Median time from randomization to surgery was 14 days in the S group. In the CS group, the median time from randomization to surgery was 63 days (36-123) for patients who received two cycles of chemotherapy, and 114 days (54-165) for patients who received additional treatment cycles. Four patients (5%) in the CS group and three patients (4%) in S group died within 30-days after surgery.

**Table 2:** Surgical details

	Total		CS group		S group		
	(N = 169)		(N = 85)		(N = 84)		*P*-value
**Surgery done**							0,083
Yes	158	(93%)	76	(90%)	82	(98%)	
No	9	(5%)	7	(8%)	2	(2%)	
Not recorded	2	(2%)	2	(2%)	0		

**Reason no surgery undertaken**							
Died before surgery	3	(2%)	1	(1%)	2	(2%)	
Progressive disease							
Tumor unresectable	3	(2%)	3	(4%)	0		
Distant metastases	3	(2%)	3	(4%)	0		

Type of resection*							0,38
Transhiatal	113	(71%)	55	(72%)	58	(71%)	
Transthoracic	20	(13%)	9	(12%)	11	(13%)	
Type not recorded	5	(3%)	4	(5%)	1	(1%)	
Other	1	(1%)	1	(1%)	0		
No resection performed	19	(12%)	7	(10%)	12	(15%)	

Postoperative deaths (within 30 days)*	7	(4%)	4	(5%)	3	(4%)	0,62

Non-fatal postoperative complications*							0,64
None	68	(43%)	30	(40%)	38	(46%)	
Any	67	(42%)	33	(43%)	34	(41%)	
Not recorded	16	(10%)	9	(12%)	7	(9%)	

Type of non-fatal postoperative complications*,^**†**^							
Pulmonary	25	(16%)	17	(23%)	8	(10%)	0,048
Cardiac	6	(4%)	3	(4%)	3	(4%)	1,0
Anastomotic							
Subclinical	10	(6%)	5	(7%)	5	(6%)	1,0
Clinical	7	(4%)	3	(4%)	4	(5%)	1,0
Chylothorax	7	(4%)	4	(5%)	3	(4%)	0,70
Bleeding	5	(3%)	3	(4%)	2	(2%)	0,67
Vocal-cord injury	22	(14%)	10	(13%)	12	(15%)	0,82
Other	10	(6%)	4	(5%)	6	(7%)	0,75

Abbreviations: *CS, Chemotherapy followed by surgery; S, Surgery alone*

* Percentages based on total patients undergoing surgery.

† Nonfatal postoperative events; comparisons were made by the Fisher's exact test

Data on postoperative complications was available of 67/76 (88%) of patients in the CS group and 75/82 (91%) patient in the S group. The frequency of nonfatal postoperative events was closely similar in both groups (table 2). However, pulmonary complications were significantly more observed in the CS group (*P *= 0.041).

Oesophagectomy was performed in 91% (69/76) in the CS-group and 85% (70/82) in the S-group. In the CS group, six patients did not receive an oesphagectomy because of tumour growth in adjacent structures (aorta or bronchial tree) and one had tumour positive celiac lymph nodes at laparotomy. In the S group, seven patients did not undergo surgical resection because of tumour encasement of the aorta or the bronchial tree and five due to tumour positive celiac lymph nodes at laparotomy. Of the 69 patients in the CS group who underwent surgical resection, 71% had R0 resections, 25% had R1 resections, and 4% had R2 resections. Of the 70 patients in the S group who underwent surgical resection, 57% had R0 resections, 29% had R1 resections, and 14% had R2 resections. Although more patients in the CS group had R0 resections as compared with the S group, no significant differences was observed (*P *= 0.09). However, there was a significant difference between the number of R2 resections in both treatment arm (*P *= 0.04). Also the number of patients with lymph node involvement (N1 or M1a) did not differ between both treatment arms (43 and 46% in the CS group and S group, respectively). In the CS group, the pathological complete response rate (pT0N0M0) was 7%.

### Pattern of failure

The outcomes of treatments were considered according to findings at operation and to patterns of disease progression (first disease-free survival event; Table 3). The rates of unresectable tumors or macroscopically incomplete resections were higher in the S group (*P *= 0.23; *P *= 0.05 respectively). The pattern of first disease progression was similar between both treatment groups; in particular there was no clear trend toward fewer patients with distant metastases as first site of relapse in the CS group. Ten patients treated with preoperative chemotherapy developed a second primary tumor; seven squamous cell carcinomas of head and neck, one pancreatic, one lung and one breast carcinoma. In contrast, four patients who underwent immediate surgical resection developed a second primary tumour, all squamous cell carcinomas of head and neck.

**Table 3:** Nature of first disease-free survival event

	CS group		S group		
Event	(N = 85)		(N = 84)		*P*-value*
Disease free	12	(14%)	7	(8%)	0,33
No surgery performed	7	(8%)	2	(3%)	0,17
No resection performed	7	(8%)	12	(14%)	0,23
Macroscopic residual disease	3	(3%)	10	(12%)	0,05
2nd Primary	10	(12%)	4	(5%)	0,16
Local recurrence	16	(19%)	21	(25%)	0,36
Distant metastases	5	(6%)	5	(6%)	1
Local recurrence and distant metastases	9	(11%)	10	(12%)	0,81
Death with cancer but site of failure not reported	5	(6%)	7	(8%)	0,57
Death from other or unspecified cause	11	(13%)	6	(7%)	0,31

* Comparisons were made by the Fisher's exact test

### Overall and disease-free survival

**Figure 2:**
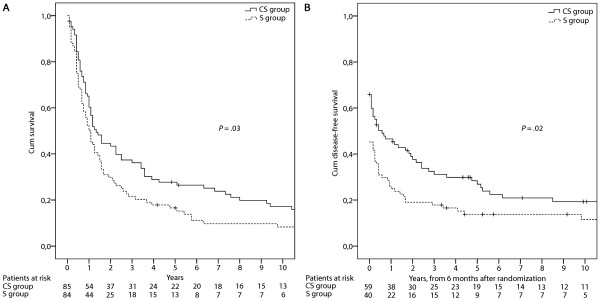
**Overall and disease free survival.** A) Overall survival of all allocated patients. The distribution curves represent the results of an intention-to-treat survival analysis involving all patients. Patients who received preoperative chemotherapy had a median survival of 16 months; in comparison, patients who underwent only surgery had a median survival of 12 months (*P *= 0.03 by the log-rank test). B) Disease-free survival of all patients from a landmark time of 6 months after date of randomisation (*P *= 0.02 by the log-rank test).

At the time of analysis, the median follow-up was 15 months in the CS group and 14 months in the S group. In an intention-to-treat survival analysis, two patients that were directly lost to follow-up were censored one month after the date of randomization. OS on intention to treat basis is shown in Figure 2. The median overall survival in the CS group was 16 months, and in the S group 12 months. OS was better in the CS group than in the S group (*P *= 0.03, by the log-rank test; HR 0.71; 95%CI 0.51-0.98; Figure 2A). Survival at one year was 64% for those allocated to chemotherapy, 52% for those allocated to surgery alone; at two years 42% and 30%; 5-years, survival was 26% and 17%, respectively.

DFS is shown in Figure 2B. For DFS, 59 patients in the CS group and 40 patients in the S group remained for analysis after the landmark period of six months. In 6/59 (10%) patients in the CS group the date of disease recurrence was not documented. In these, the date of disease recurrence was estimated four months earlier than the date of death (in the CS group the median time between date of recurrence and date of death was four months). In the CS group, there is prolonged DFS compared with the surgical resection alone group (*P *= 0.02, by the log-rank test; HR 0.72; 95%CI 0.52-1.0).

Overall survival according clinical response to preoperative chemotherapy showed that patients with clinical partial or complete response (those who received three or more cycles of therapy) had significantly better overall survival then those with stable or progressive disease (*P *= <0.001, by log-rank test; HR 0.38; 95%CI 0.23-0.65).

**Figure 3:**
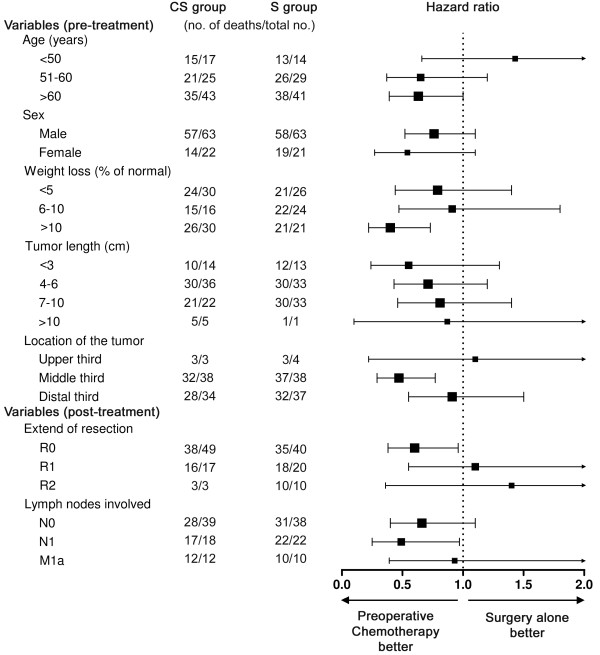
**Survival by characteristics at randomisation and post-treatment**. Centre of each square indicates hazard ratio, and area of square the amount of information. Lines on either side indicate 95 CI.

Figure 3 shows no clear evidence that effect of chemotherapy varied in accordance with age, sex or length of the tumour. In this subgroup analysis, it appeared that patients with substantial weight loss (>10%) treated with preoperative chemotherapy had better overall survival as compared to those who received surgery alone. Possibly, patients allocated to chemotherapy and in a poor nutrition status (eg weight loss >10%) were more likely to receive nutritional support over a longer period of time as compared to patients that were allocated to surgery alone. This could have led to a better preoperative condition of patients who received chemotherapy, which could possibly contribute to improved overall survival. Furthermore, patients with a tumour located in the middle thoracic oesophagus who received preoperative chemotherapy had better overall survival then patients who received surgery alone. The explanation for the observed survival benefit in this subgroup of patients is unclear.

## Discussion

The long-term results of this randomized controlled trial demonstrated a survival benefit for preoperative chemotherapy followed by surgery in patients with OSCC, when compared with surgery alone. This study has only been reported in abstract form, which hampers interpretation of our findings in context of other randomized trials [9]. Why it took so long to report the design and results of this study is not completely understood. The main reasons are change of personnel (the trial coordinator [TCK] moved to another hospital) and loss of interest in the used chemotherapeutic regime. Nevertheless, we believe that these results contribute to the ongoing debate about the optimal (preoperative) therapy for patients with OSCC.

The results of this study should be interpreted in the timeframe in which this study was performed. This study is one of the three largest randomized controlled trials among patients with OSCC treated with preoperative chemotherapy followed by surgery or surgery alone [10,11]. All these large randomized controlled trials were performed in the early '90s. The Medical Research Council (MRC) trial included most patients with oesophageal cancer (533 oesophageal adenocarcinoma (OAC) and 247 OSCC patients) and demonstrated a significant survival benefit for the use of preoperative chemotherapy (P = .004) [11,12]. An other large and well-conducted randomized controlled trial among patients with oesophageal cancer (236 OAC and 204 OSCC patients), by the North American Intergroup (RTOG Trial 8911 or USA Intergroup 113 trial; further called the Intergroup trial), demonstrated no significant difference in survival between patients treated with preoperative chemotherapy and those who received surgery alone [10,13]. In the light of the results of both trials, we discuss the design and results of the present study.

As most randomized controlled trials performed in the early '90, this study reflects the methods for diagnosis, staging, treatment delivery and outcome measurement that indicate clinical practice during that period. In the present study, the majority of patients (88%) underwent preoperative staging by oesophago-gastroscopy and CT scan of the chest and upper abdomen. The same preoperative staging methods were used in the Intergroup trial. In the MRC trial, however, there was no standardization of preoperative staging. These differences in preoperative staging could, by selection of different populations of OSCC patients, contribute to differences in results between the trials.

In the Intergroup trial as well as in the MRC trial the chemotherapeutic regime consists of cisplatin combined with fluorouracil; in the present study cisplatin was combined with etoposide. The ratio for this combination of chemotherapeutic agents was deducted from trials among patients with non-small-cell lung cancer in which this regime had showed to be safe and effective [14]. Furthermore, a phase II trial in patients with advanced OSCC had shown that the response rate equals that of other cisplatin-based regimes and that the toxicity profile was mild [8]. Patients without clinical response to chemotherapy received a total preoperative dose of 160 mg/m^2 ^cisplatin and 1000 mg/m^2 ^etoposide. The dose of cisplatin is similar as compared with the MRC trial (160 mg/m^2^). Patients with clinical response to chemotherapy received total doses up to cisplatin 320 mg/m^2 ^and etoposide 2000 mg/m^2^. In this subgroup of patients, the total preoperative dose of cisplatin was slightly higher as compared to the Intergroup trial (300 mg/m^2^). The compliance to chemotherapy was 88% (patients who received two or more of the planned cycles of chemotherapy). This is similar to 90% of the patients that received both treatment cycles in the MRC trial, but differed from the Intergroup wherein 71% of the patients received all of three planned cycles of chemotherapy. It has been suggested that the higher dose of chemotherapy in the Intergroup trial was detrimental to patients who underwent oesophagectomy. Other factors related to the chemotherapeutic regimes that could contribute to the differences in outcome between the three studies, are the use of chemoradiotherapy in a subset of patients in the MRC trial and the use of postoperative chemotherapy in a subgroup of patients in the Intergroup trial.

In our study the majority of patients underwent a transhiatal oesophagectomy. This type of resection is associated with lower morbidity (and mortality) than a transthoracic resection ([15]). In the other trials, the type of surgical resection that has been performed is not clear (MRC trial) or the exact numbers are not described (Intergroup trial). The postoperative mortality rate (<30 days after surgery) in the current trial was 5% (4/76) in the CS group and 4% (3/82) in the S group and differed not among both groups. These rates are similar as those observed in the Intergroup trial, with 6% postoperative mortality in both treatment arms. In contrast, the MRC trial reported much higher postoperative mortality rate of 10% in the CS group and 11% in the S group.

In the present study surgery was performed in 89% and 98% of patients in the CS group and S group, respectively. Similar rates have been reported by the MRC trial, with surgery rates in the CS group of 92% and in the S group of 97%. In the Intergroup trial fewer patients underwent surgery, 80% of the CS group and 92% of the S group. The rate of microscopically tumour free resection margins (R0) in the CS group was 71%, as compared to 60% and 62% in the CS groups of the MRC and Intergroup trial, respectively. In the S group it was 57%, as compared to 54% and 59% in the S groups of the MRC and Intergroup trial, respectively. The difference in R0 resection rates between the CS group and the S group is likely to contribute to the observed survival benefit for patients treated with preoperative chemotherapy (as showed by the MRC trial; *P *< 0.001), however, this difference was not statistical significant in the present study (*P *= 0.086).

The median survival time of the CS group was 16 months, compared to 17 and 15 months in the MRC and Intergroup trial, respectively. The median survival time of the S group was 12 months, compared to 16 and 13 months in the Intergroup and MRC trial, respectively. It appears that the S group in our study had the worst survival outcome, but this may be due to patient selection. Both the MRC as Intergroup trial included more OAC than OSCC patients. Subgroup analysis of the MRC trial, including only OSCC patients, showed a median survival time of 11 months for patients who underwent surgery alone [12]. Remarkably, in the subgroup analysis there is no significant survival benefit for OSCC patients treated with preoperative chemotherapy (P = 0.1).

In line with the results of the MRC and Intergroup trial, there was no significant difference in pattern of failure between both treatment arms in our study. The rate of distant metastases was equal in both treatment groups. These findings provide no evidence for the general hypothesis that preoperative chemotherapy eliminates systemic micro-metastases. The results of this trial and the MRC trial suggest that the biologic effect of preoperative chemotherapy seems to specifically influence the extent of surgery [12]. In the present study, the incidence of incomplete resections was greater in the S group, but sites of first recurrence (local or distant) were similar. Furthermore, at 6 months, the DFS advantage is established for the CS group (Figure 2A) and remains consistent throughout follow-up as the survival curves remain approximately parallel. This suggests that the effect of preoperative chemotherapy is to reduce tumour volume and increase the potential for curative resection.

This study has its limitations. At first, the preoperative staging was hampered by the absence of endoscopic ultrasonography at the beginning of our trial. Therefore, the clinical T- and N-stage were not used as stratification parameters before randomization. Secondly, the missing data on two patients that underwent preoperative chemotherapy and the lack of some clinical characteristics of the patients reflect the difficulty of obtaining all data more than twenty years after the trial was performed. At third, it should be noticed that we selected patients who showed clinical response to chemotherapy based on oesophago-gastroscopy and CT scan of the chest and upper abdomen, for additional cycles of chemotherapy. However, we did not correlate clinical response to pathological response. Therefore, selection of this subgroup could also reflect better prognostic characteristics of patients who respond to chemotherapy, rather than an effect of chemotherapy itself.

## Conclusions

In summary, this study reports a significant survival benefit for OSCC patients treated with preoperative chemotherapy. The chemotherapeutic regime used in this trial (etoposide and cisplatin) is not used anymore in treatment of patients with OSCC. Today, in our institution (EMC) the majority of patients with OSCC (and OAC) receive preoperative chemoradiotherapy (a combination of carboplatin and paclitaxel, and concurrent radiotherapy).

## Abbreviations

OSCC: oesophageal squamous cell carcinoma; S-group: patients who were randomly assigned to undergo surgery alone; CS-group: patients who were randomly assigned to preoperative chemotherapy; OS: overall survival; DFS: disease free survival; CT: computed tomography; WHO: world health organisation; CR: complete response; PR: partial response; SD: stable disease; R0: microscopical tumour free resection margins; R1: microscopical tumour-positive circumferential margin; R2: macroscopical residual disease; EMC: erasmus medical center; HR: hazard ratio; MRC: medical research council; OAC: oesophageal adenocarcinoma.

## Competing interests

The authors declare that they have no competing interests.

## Authors' contributions

JJB made substantial contributions to data collection, data analysis, and drafted the manuscript. TCK was responsible for conception and design of the trial and coordinated the study. MH and FJWK participated in data collection. BPLW, MIBH and WNMD were involved in revising the manuscript for important intellectual content. PDS, JJBL and HWT participated in the design of the study and revised the manuscript. AG made substantial contributions to data collection, data analysis, and revised the manuscript. All authors have read an approved the final version of the manuscript.

## Pre-publication history

The pre-publication history for this paper can be accessed here:

http://www.biomedcentral.com/1471-2407/11/181/prepub
